# SARS-CoV-2 Inhibits NRF2-Mediated Antioxidant Responses in Airway Epithelial Cells and in the Lung of a Murine Model of Infection

**DOI:** 10.1128/spectrum.00378-23

**Published:** 2023-04-06

**Authors:** Yue Qu, Aline Haas de Mello, Dorothea R. Morris, Yava L. Jones-Hall, Teodora Ivanciuc, Rachel A. Sattler, Slobodan Paessler, Vineet D. Menachery, Roberto P. Garofalo, Antonella Casola

**Affiliations:** a Department of Pediatrics, The University of Texas Medical Branch, Galveston, Texas, USA; b Department of Microbiology and Immunology, The University of Texas Medical Branch, Galveston, Texas, USA; c School of Veterinary Medicine and Biomedical Sciences, Texas A&M University, College Station, Texas, USA; d Department of Pathology, The University of Texas Medical Branch, Galveston, Texas, USA; University of Siena

**Keywords:** SARS-CoV-2, COVID-19, NRF2, antioxidant enzymes, *Nrf2*-deficient mice

## Abstract

Several viruses have been shown to modulate the transcription factor nuclear factor erythroid 2-related factor 2 (NRF2), the master regulator of redox homeostasis. The severe acute respiratory syndrome coronavirus 2 (SARS-CoV-2), responsible for the COVID-19 pandemic, also seems to disrupt the balance between oxidants and antioxidants, which likely contributes to lung damage. Using *in vitro* and *in vivo* models of infection, we investigated how SARS-CoV-2 modulates the transcription factor NRF2 and its dependent genes, as well as the role of NRF2 during SARS-CoV-2 infection. We found that SARS-CoV-2 infection downregulates NRF2 protein levels and NRF2-dependent gene expression in human airway epithelial cells and in lungs of BALB/c mice. Reductions in cellular levels of NRF2 seem to be independent of proteasomal degradation and the interferon/promyelocytic leukemia (IFN/PML) pathway. Furthermore, lack of the *Nrf2* gene in SARS-CoV-2-infected mice exacerbates clinical disease, increases lung inflammation, and is associated with a trend toward increased lung viral titers, indicating that NRF2 has a protective role during this viral infection. In summary, our results suggest that SARS-CoV-2 infection alters the cellular redox balance by downregulating NRF2 and its dependent genes, which exacerbates lung inflammation and disease, therefore, suggesting that the activation of NRF2 could be explored as therapeutic approach during SARS-CoV-2 infection.

**IMPORTANCE** The antioxidant defense system plays a major function in protecting the organism against oxidative damage caused by free radicals. COVID-19 patients often present with biochemical characteristics of uncontrolled pro-oxidative responses in the respiratory tract. We show herein that SARS-CoV-2 variants, including Omicron, are potent inhibitors of cellular and lung nuclear factor erythroid 2-related factor 2 (NRF2), the master transcription factor that controls the expression of antioxidant and cytoprotective enzymes. Moreover, we show that mice lacking the *Nrf2* gene show increased clinical signs of disease and lung pathology when infected with a mouse-adapted strain of SARS-CoV-2. Overall, this study provides a mechanistic explanation for the observed unbalanced pro-oxidative response in SARS-CoV-2 infections and suggests that therapeutic strategies for COVID-19 may consider the use of pharmacologic agents that are known to boost the expression levels of cellular NRF2.

## INTRODUCTION

Viruses can cause disease by replicating and killing host cells as well as by triggering excessive local or systemic inflammatory responses. In addition, viruses induce oxidative stress and tissue injury as a major pathogenetic mechanism of disease (1). This process has been shown, for example, in human respiratory infections caused by respiratory syncytial virus (RSV), human metapneumovirus (hMPV), and influenza virus (2–5). The antioxidant enzymes (AOEs) play a major role in protecting the organism against oxidative damage caused by reactive oxygen species (ROS) and reactive nitrogen species (RNS) (6–9). Transcription of many oxidative stress-inducible genes is regulated in part through *cis*-acting antioxidant responsive element (ARE) sequences. This element has been identified in the regulatory regions of genes involved in the antioxidant response and detoxification processes (10, 11), such as NAD(P)H quinone dehydrogenase 1 (*NQO1*), heme oxygenase 1 (*HMOX1*), superoxide dismutases (*SOD1* to *-3*), catalase (*CAT*), glutathione peroxidases (*GPX1* to -*8*), glutathione *S*-transferases (GSTs), glutamate-cysteine ligase catalytic subunit (*GCLC*) and modifier subunit (*GCLM*), and peroxiredoxins (*PRDX1* to -*6*) (1, 12–14).

The nuclear factor erythroid 2-related factor 2 (NRF2) is an important redox-responsive protein that helps to protect cells from oxidative stress and damage. NRF2 is a transcription factor that under homeostatic/unstressed conditions is bound in the cytosol to a cytoskeleton-associated inhibitor called kelch-like ECH-associated protein 1 (KEAP1). Electrophile-induced release of NRF2 is proposed to involve covalent modifications of KEAP1 and/or NRF2. The released NRF2 then translocates to the nucleus and binds to ARE sites to promote gene transcription (10, 15, 16). Besides being a key regulator of redox homeostasis, NRF2 also has a role in the regulation of antiviral interferon (IFN) responses and can contribute to resolution of inflammation and tissue repair (11, 17).

Viruses have several strategies to manipulate the host cell machinery to their advantage and to maintain favorable cellular conditions for their replication (12, 18). In addition to ROS production, some viruses have been shown to target the host antioxidant response, by either activating or inhibiting NRF2 (1, 11, 12, 17). The activation or downregulation of NRF2 depends on the type of virus and the phase of the replicative cycle (19). Activation of the NRF2 signaling pathway induces cytoprotective genes and maintains cellular homeostasis for cell survival and continuous replication of the virus, contributing to chronic and persistent viral infections (12). On the other hand, by inhibiting NRF2 activation or increasing its degradation, viral infections can cause a progressive decrease in the expression of AOEs, leading to the accumulation of reactive species and subsequent cellular damage and lung injury (5).

Recent reports have shown that infection with the severe acute respiratory syndrome coronavirus 2 (SARS-CoV-2), the causative agent of coronavirus disease 2019 (COVID-19), is associated with the production of oxidative stress markers, which may be of predictive value for disease severity in infected patients (17, 20–24). In addition, a study with differential gene expression analysis using publicly available transcriptome data sets of lung biopsy specimens from adult COVID-19 patients has shown that, while genes linked with inflammatory and antiviral pathways, including RIG-I receptor and Toll-like receptor (TLR) signaling were enriched in these samples, genes associated with the NRF2-dependent antioxidant response were suppressed in the same patients (25). Based on these published observations, we investigated in this study the effect of SARS-CoV-2 infection on the NRF2 cellular levels and NRF2-dependent gene expression in human airway epithelial cells and in the lungs of mice. Furthermore, the role of NRF2 in SARS-CoV-2 experimental infection and lung pathology was determined in mice genetically deficient in *Nrf2*. The results of this study suggest that therapeutic strategies for COVID-19 may consider the use of new pharmacologic agents or repurposing of pharmacologic agents that are known to boost the expression levels of cellular NRF2.

## RESULTS

### SARS-CoV-2 infection downregulates NRF2 protein levels in epithelial cell lines, including human lung-derived cells.

**FIG 1:**
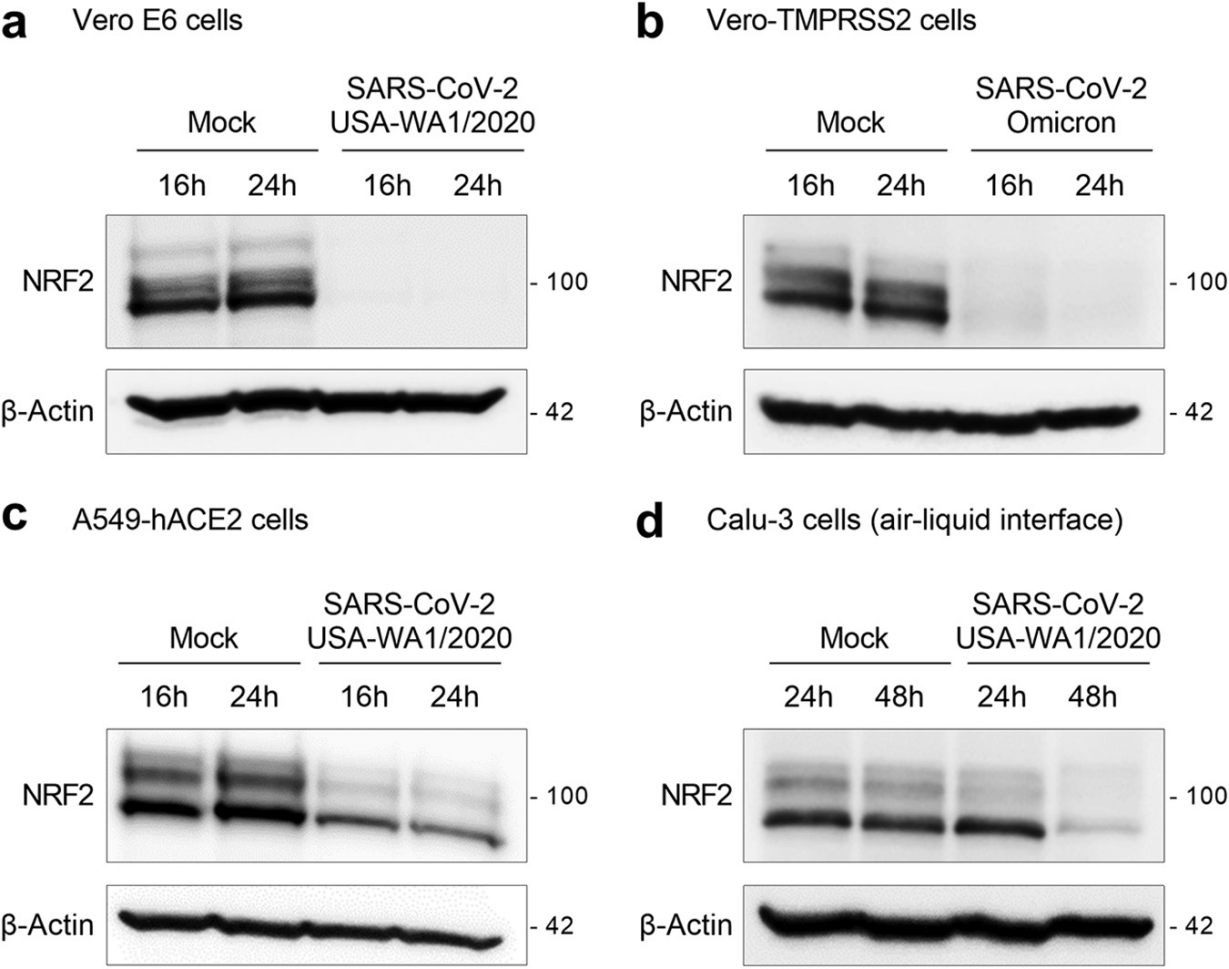
SARS-CoV-2 infection downregulates NRF2 protein levels in epithelial cell lines, including human lung-derived cells. (a) Vero E6 cells were infected with SARS-CoV-2 USA-WA1/2020, (b) Vero-TMPRSS2 cells were infected with the SARS-CoV-2 Omicron (B.1.1.529) variant, and (c) A549-hACE2 cells were infected with SARS-CoV-2 USA-WA1/2020. Cells were harvested at 16 and 24 h postinfection (hpi), and whole-cell lysates were analyzed by Western blotting with anti-NRF2 antibody. The membranes were reprobed for anti-β-actin antibody for a loading control. Western blot images are one representative of two independent experiments. (d) Calu-3 cells cultured on an air-liquid interface (ALI) were infected with SARS-CoV-2 USA-WA1/2020. Cells were harvested at 24 and 48 hpi, and whole-cell lysates were analyzed by Western blotting with anti-NRF2 antibody. The membrane was reprobed for anti-β-actin antibody for a loading control. Data from one experiment performed in triplicate are shown.

To determine how SARS-CoV-2 infection affects the cellular levels of the transcription factor NRF2, we selected two strains of SARS-CoV-2: the early pandemic USA-WA1/2020 (here, WA1) strain and the more recent Omicron variant (B.1.1.529). In the first experiments, Vero E6 cells were infected with SARS-CoV-2 WA1, harvested 16 and 24 h postinfection (hpi), and whole-cell lysates were analyzed by Western blotting with anti-NRF2 antibody. As shown in Fig. 1a, infection with SARS-CoV-2 WA1 induced a dramatic decrease in NRF2 levels at both time points, compared to uninfected cells (mock). Similarly, NRF2 protein levels were also strikingly decreased in Vero cells stably expressing human transmembrane serine protease 2 (Vero-TMPRSS2) and infected with SARS-CoV-2 B.1.1.529 (Fig. 1b). Given the similar results observed with both viruses, all subsequent experiments *in vitro* were performed with the USA-WA1/2020 strain.

Although Vero cells are naturally permissive to SARS-CoV-2 infection and commonly used for virus propagation (26, 27), they may not represent the most appropriate model of SARS-CoV-2-infected airway epithelial cells. Thus, we performed the following experiments in human lung-derived epithelial cell lines. A549 cells (type II-derived epithelial cells) stably expressing human angiotensin-converting enzyme 2 (ACE2) receptor (A549-hACE2) (28) were infected for 16 and 24 h, and whole-cell lysates were analyzed for NRF2 by Western blotting. As shown in Fig. 1c, SARS-CoV-2 induced a considerable reduction in NRF2 protein levels in A549-hACE2 cells compared to uninfected cells, albeit not to the same extent as in Vero cells. We next evaluated the NRF2 protein levels after SARS-CoV-2 infection on epithelial cells cultured on an air-liquid interface (ALI), a more physiologically relevant model of airway epithelium differentiation. For that, we selected Calu-3 cells, which are naturally susceptible to SARS-CoV-2 infection (29). Western blot analysis corroborated the results with the other cell lines, by demonstrating a great reduction in the NRF2 protein levels at the 48-h time point (Fig. 1d). Taken together, these results demonstrate that NRF2 protein levels are downregulated in multiple epithelial cells after SARS-CoV-2 infection, including in human lung-derived epithelial cells, grown under either standard submerged cell culture or ALI conditions.

### SARS-CoV-2 infection downregulates NRF2-dependent gene expression in human lung epithelial cells.

**FIG 2:**
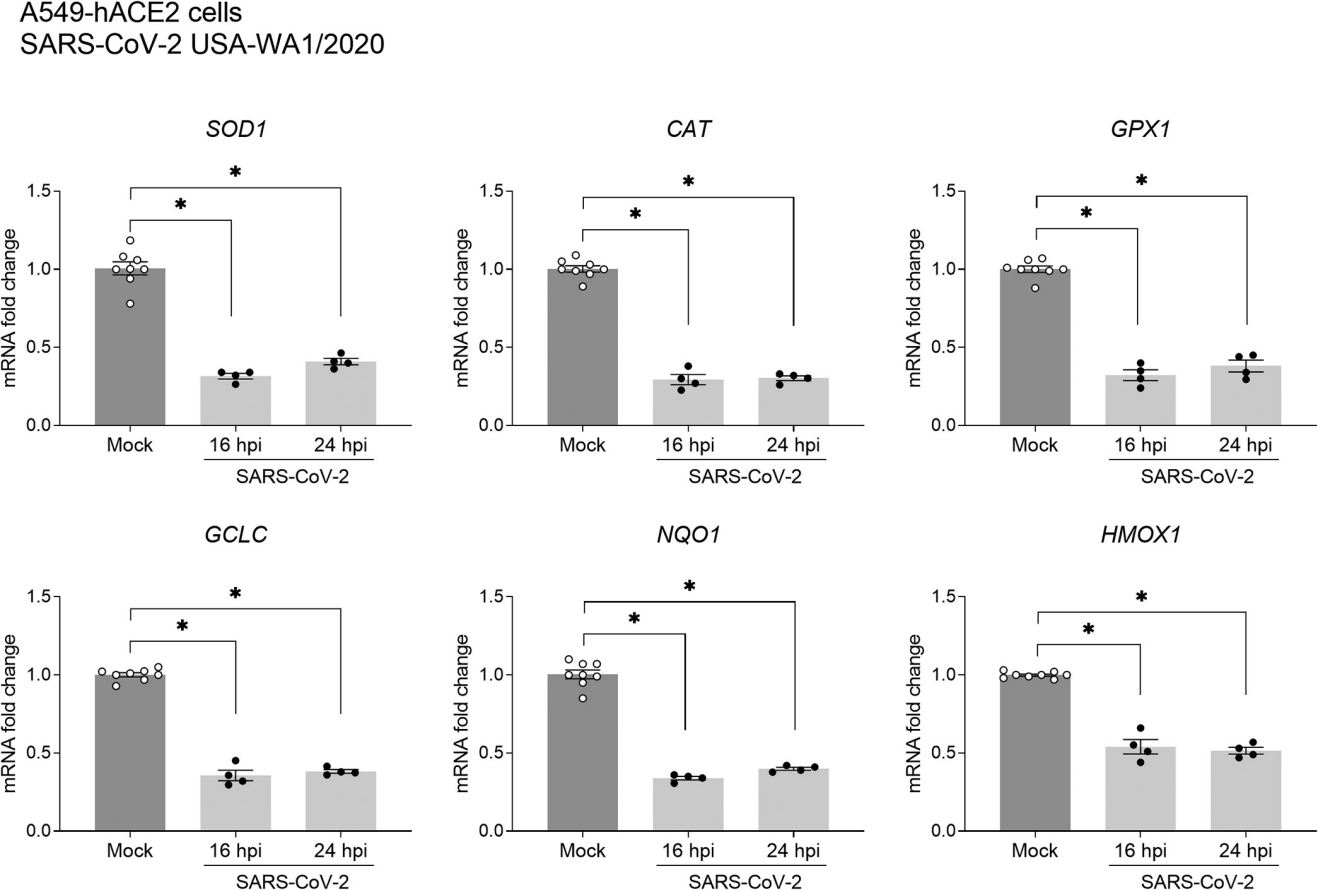
SARS-CoV-2 infection downregulates NRF2-dependent gene expression in human lung epithelial cells. A549-hACE2 cells mock infected or infected with SARS-CoV-2 (USA-WA1/2020) were harvested 16 and 24 hpi to prepare total RNA. *SOD1*, *CAT*, *GPX1*, *GCLC*, *NQO1*, and *HMOX1* gene expression was quantified by RT-qPCR. The graphs show combined data from two independent experiments expressed as mean ± SEM. Data were analyzed by one-way analysis of variance (ANOVA) followed by Tukey’s test (*, *P* < 0.05).

Given the role of NRF2 in the regulation of several antioxidant and cytoprotective genes, A549-hACE2 cells were infected with SARS-CoV-2 WA1, total RNA was isolated, and *SOD1*, *CAT*, *GPX1*, *GCLC*, *NQO1*, and *HMOX1* gene expression was analyzed by real-time quantitative PCR (RT-qPCR). As shown in Fig. 2, SARS-CoV-2 infection induced a significant reduction of all genes analyzed, an indication of the critical function of NRF2 in controlling the cellular levels of AOEs and the susceptibility of this innate pathway to the inhibitory effect of SARS-CoV-2 infection.

### Decreased NRF2 protein expression is independent of proteasomal degradation and the interferon/promyelocytic leukemia pathway.

**FIG 3:**
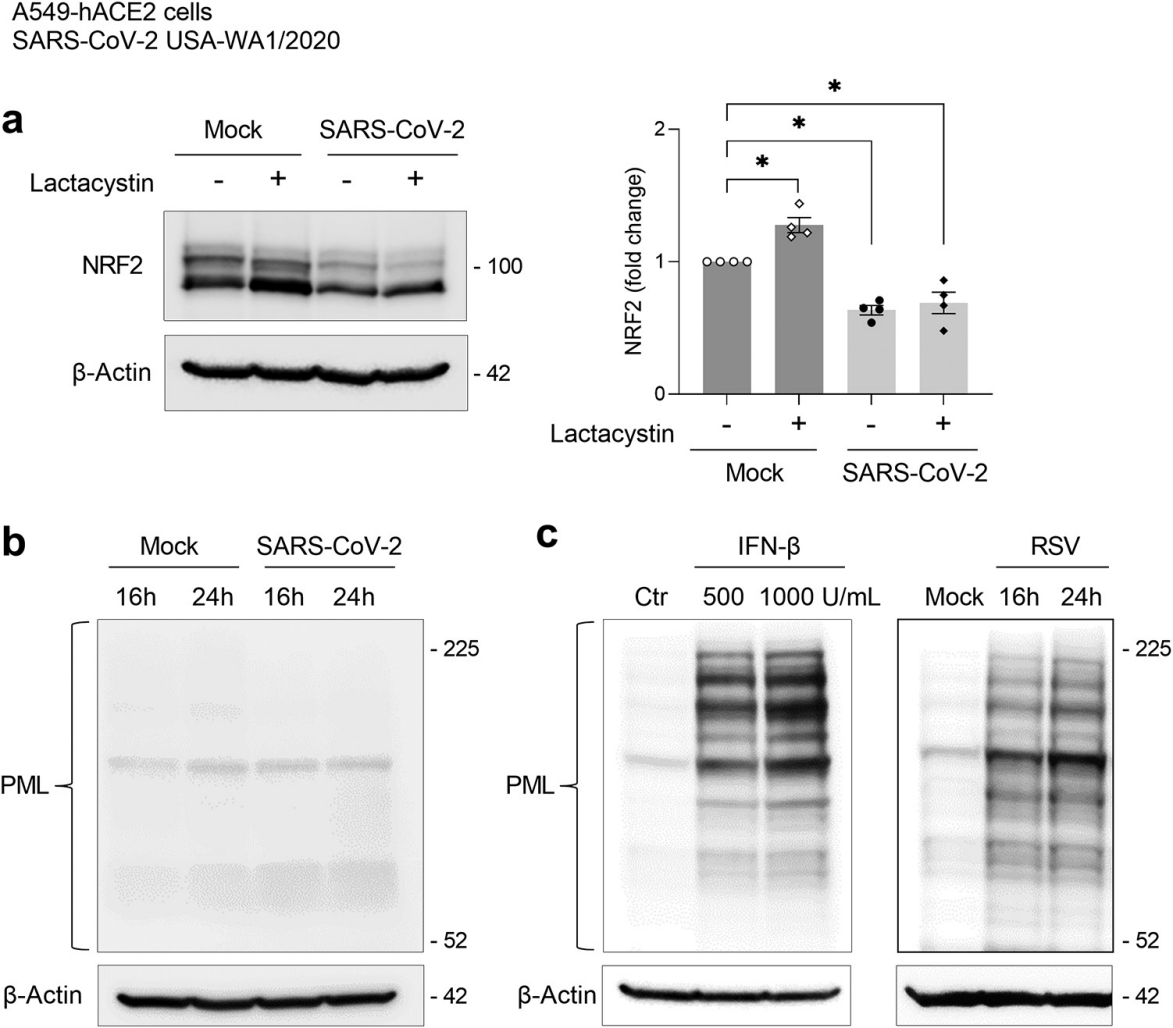
NRF2 decreased cellular levels are independent of proteasomal degradation and the interferon/promyelocytic leukemia (IFN/PML) pathway. (a) A549-hACE2 cells mock infected or infected with SARS-CoV-2 (USA-WA1/2020) were treated with 10 μM lactacystin or its vehicle (at 6 hpi) and harvested 20 hpi. Whole-cell lysates were analyzed by Western blotting with anti-NRF2 antibody. The membranes were reprobed for anti-β-actin antibody for loading control. The Western blot image is one representative of four independent experiments. The graph shows the densitometric analysis of NRF2 after normalization to β-actin expressed as mean ± SEM (*, *P* < 0.05 by two-way ANOVA followed by Tukey’s test). (b) Whole-cell lysates from A549-hACE2 cells mock infected or infected with SARS-CoV-2 for 16 and 24 h were analyzed by Western blotting with anti-PML protein antibody. (c) Whole-cell lysates from A549-hACE2 cells untreated and treated with human IFN-β for 16 h and mock infected or infected with RSV for 16 and 24 h were analyzed by Western blotting with anti-PML protein antibody. The membranes were reprobed for anti-β-actin antibody for a loading control. Western blot images are one representative of two independent experiments.

We have previously shown that RSV, a common respiratory virus, downregulates NRF2 levels in infected cells by increasing NRF2 ubiquitination and proteasomal degradation, and blocking proteasome-mediated degradation rescues NRF2 cellular levels (30). Therefore, to determine whether a similar mechanism also occurred in SARS-CoV-2 infection, A549-hACE2 cells were treated with the proteasome inhibitor lactacystin during infection. Western blot analysis of whole-cell lysates showed that lactacystin did not rescue NRF2 levels after SARS-CoV-2 infection (Fig. 3a), suggesting that reduced NRF2 expression caused by SARS-CoV-2 infection is not due to increased proteasomal degradation. On the other hand, uninfected (mock infected) cells treated with lactacystin showed some modest increase of NRF2, an expected effect of proteasome inhibition.

NRF2 can also be degraded through other mechanisms. In previous studies, we found that RSV infection causes upregulation of promyelocytic leukemia (PML) protein and leads to NRF2 degradation via the SUMOylation-dependent ubiquitin ligase RING finger protein 4 (RNF4) associated with PML nuclear bodies (NBs), which are induced via an IFN-dependent pathway in infected epithelial cells (31). To determine whether the decreased NRF2 levels following SARS-CoV-2 could be also related to increased PML protein expression after infection, whole-cell lysates of SARS-CoV-2-infected A549-hACE2 cells were analyzed by Western blotting using anti-PML antibody. As shown in Fig. 3b, SARS-CoV-2 infection did not upregulate PML protein levels, while treatment of the cells with human IFN-β and or infection with RSV resulted in a robust induction of PML (Fig. 3c). These results suggest that mechanisms distinct from those of other viral respiratory pathogens are involved in SARS-CoV-2-mediated NRF2 downregulation.

### SARS-CoV-2 infection downregulates NRF2-dependent gene expression in the lungs of mice.

**FIG 4:**
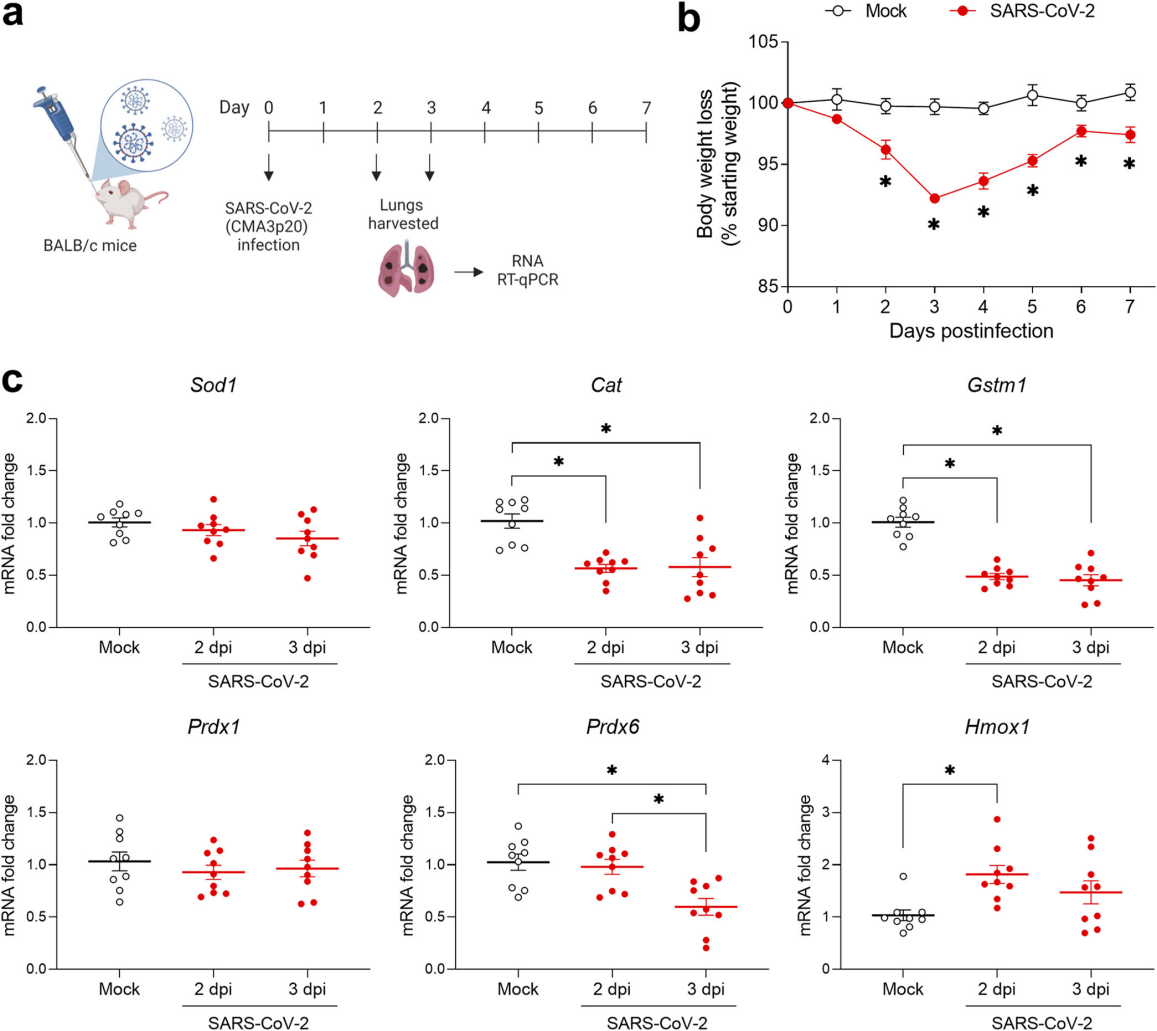
SARS-CoV-2 infection downregulates NRF2-dependent gene expression in lungs of BALB/c mice. (a) Eleven- to 12-week-old female BALB/c mice were inoculated intranasally with 5 × 10^6^ to 10^7^ TCID_50_s of mouse-adapted SARS-CoV-2 (CMA3p20) or mock inoculated with PBS. The schematic figure was created with BioRender.com. (b) Body weight loss was monitored for 7 days to examine the kinetics of the disease. Data are expressed as mean ± SEM (*n *= 3 or 4 mice/group; *, *P* < 0.05 by Student’s *t* test). (c) A group of mice were euthanized at 2 and 3 days postinfection (dpi), and lungs were harvested to isolate total RNA. *Sod1*, *Cat*, *Gstm1*, *Prdx1*, *Prdx6*, and *Hmox1* gene expression was quantified by RT-qPCR. Data are expressed as mean ± SEM (*n *= 9 mice/group; one-way ANOVA followed by Tukey’s test: *, *P* < 0.05).

We next investigated whether SARS-CoV-2 infection would affect the NRF2-dependent antioxidant pathway in the lung. After confirming that the mouse-adapted strain of SARS-CoV-2 CMA3p20 (32) still preserves the ability to downregulate NRF2 (see Fig. S1 in the supplemental material), groups of adult female BALB/c mice were infected with SARS-CoV-2 CMA3p20 or inoculated with the same volume of vehicle/phosphate-buffered saline (PBS) (mock infected) (Fig. 4a). In the initial experiment, mice were monitored daily for body weight loss as measure of clinical disease, up to day 7 when infection by CMA3p20 is cleared (32) (Fig. 4b). In the following experiments, mice were euthanized at 2 and 3 days postinfection (dpi) (the peak of body weight loss), and lungs were excised for analysis of NRF2-dependent genes (*Sod1*, *Cat*, *Gstm1*, *Prdx1*, *Prdx6*, and *Hmox1)* by RT-qPCR. As shown in Fig. 4c, CMA3p20-infected mice had significantly lower expression of *Cat* and *Gstm1* at 2 and 3 dpi and *Prdx6* at 3 dpi in the lung compared to mock-inoculated mice. *Sod1* and *Prdx1* were not significantly affected by infection, while interestingly *Hmox1* expression was increased at 2 dpi (Fig. 4c).

### Genetic deficiency of *Nrf2* exacerbates disease and causes a modest increase in lung inflammation in mice infected with SARS-CoV-2.

**FIG 5:**
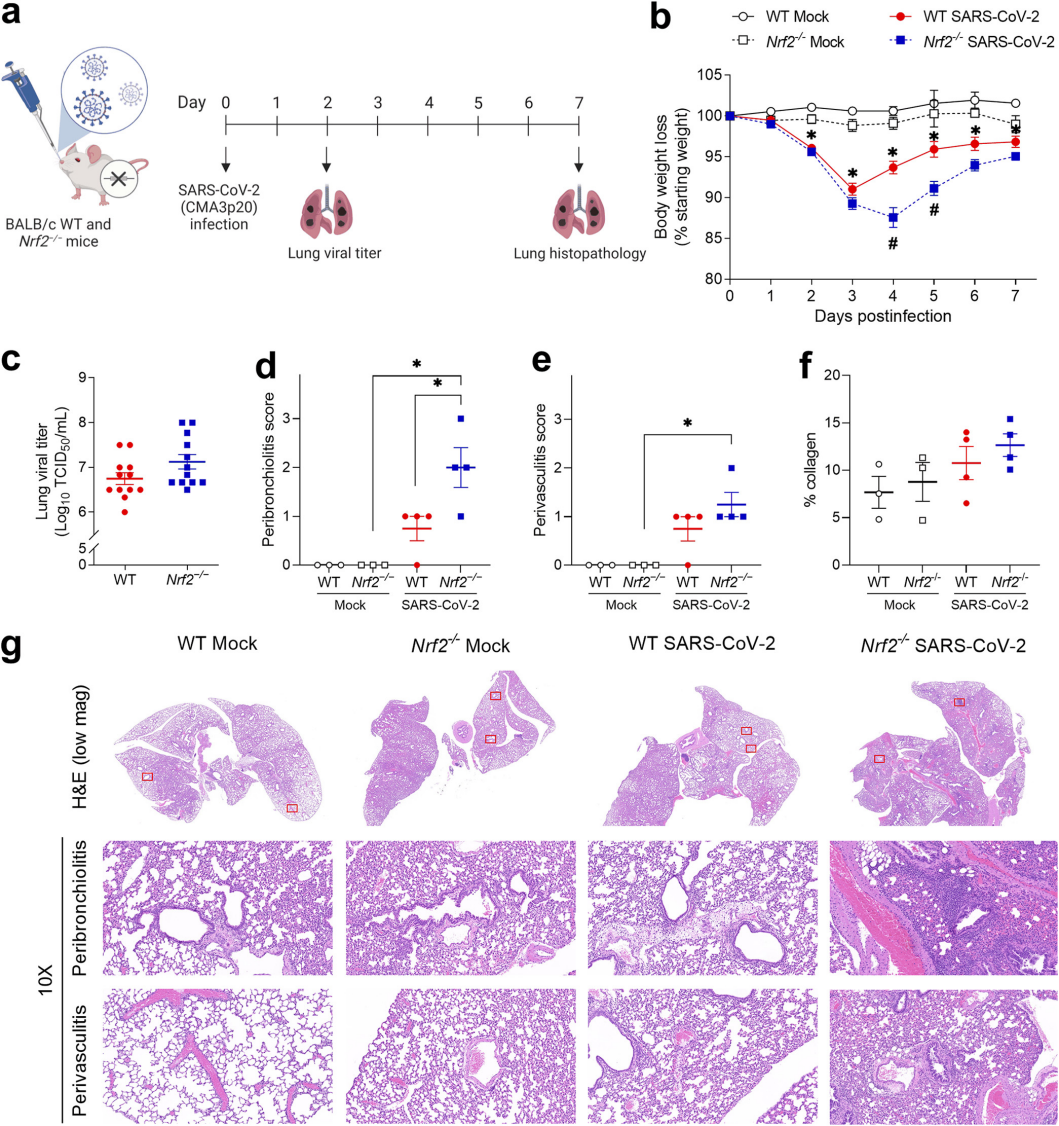
Lack of the *Nrf2* gene in mice exacerbates clinical disease and peribronchiolitis following SARS-CoV-2 infection. (a) Sixteen- to 20-week-old *Nrf2^−/−^* and wild-type (WT) age-matched BALB/c female mice were infected with 10^6^ TCID_50_s of mouse-adapted SARS-CoV-2 (CMA3p20) or mock inoculated with PBS. Schematic figure created with BioRender.com. (b) Changes in body weight were monitored for 7 days. Data are expressed as mean ± SEM (mock, *n *= 3 mice/group; SARS-CoV-2, *n *= 10 to 12 mice/group; *, *P* < 0.05 for WT mock infected versus WT SARS-CoV-2, and *#*, *P* < 0.05 for WT SARS-CoV-2 versus *Nrf2^−/−^* SARS-CoV-2, by two-way ANOVA followed by Tukey’s test). (c) Lung viral titers at 2 dpi were determined by TCID_50_ assay. Data are expressed as mean ± SEM (*n *= 12 mice/group; *P* = 0.08 by Student’s *t* test). (d) Peribronchiolitis and (e) perivasculitis scored at 7 dpi in hematoxylin and eosin (H&E) stained sections of lung; (f) percentage of collagen in lung tissue at 7 dpi determined in Masson’s trichrome-stained sections as described in Materials and Methods. Data are expressed as mean ± SEM (*n *= 3 or 4 mice/group; *, *P* < 0.05 by two-way ANOVA followed by Tukey’s test). (g) Representative images of H&E-stained sections of lung depicting inflammation surrounding bronchioles and vessels (subgross images at ×1.0 and insets at ×10 magnification).

To better understand the role of NRF2 in the context of SARS-CoV-2 infection, mice genetically deficient in *Nrf2* (*Nrf2^−/−^*) on the BALB/c background and wild-type (WT) controls were infected with CMA3p20 or mock inoculated (Fig. 5a). Mock-inoculated WT and *Nrf2^−/−^* mice did not exhibit any signs of disease or weight loss over a 7-day period (Fig. 5b). Both infected WT and *Nrf2^−/−^* mice lost body weight during the observation period, but *Nrf2^−/−^* mice had signs of exacerbated clinical disease, as shown by significant greater weight loss at 4 and 5 dpi, compared to WT mice (Fig. 5b). In addition, recovery from weight loss was delayed in *Nrf2^−/−^* mice, while in WT mice weight loss peaked at 3 dpi and they started to recover at day 4: *Nrf2^−/−^* mice were still losing weight at day 4 and started to recover only after 5 dpi.

To determine whether *Nrf2* deficiency would affect viral replication in the lung, infected *Nrf2^−/−^* and WT control mice were euthanized at 2 dpi, which was previously reported to be the peak viral replication in the lungs of BALB/c mice infected with CMA3p20 (32). Lung viral titers were determined by a 50% tissue culture infective dose (TCID_50_) assay. As shown in Fig. 5c, infected *Nrf2^−/−^* mice exhibited a trend toward higher viral titers in the lung than WT control mice (*P* = 0.08).

To assess lung histopathology, groups of mice were euthanized 7 dpi and lungs were collected. Analysis of hematoxylin and eosin (H&E)-stained lung sections showed significantly greater cellular infiltration surrounding the bronchioles (peribronchiolitis score) in infected *Nrf2^−/−^* mice compared to infected WT mice, but there were no significant differences between these groups in cellular infiltration surrounding vessels (perivasculitis) (Fig. 5d, e, and g). Although this was a relatively early time point, Masson’s trichrome-stained lungs sections did not show significant differences in pulmonary fibrosis between infected mice and to mock-inoculated ones; however, there was a trend for higher percentage of collagen staining in infected *Nrf2^−/−^* mice compared to infected WT mice (Fig. 5f).

**FIG 6:**
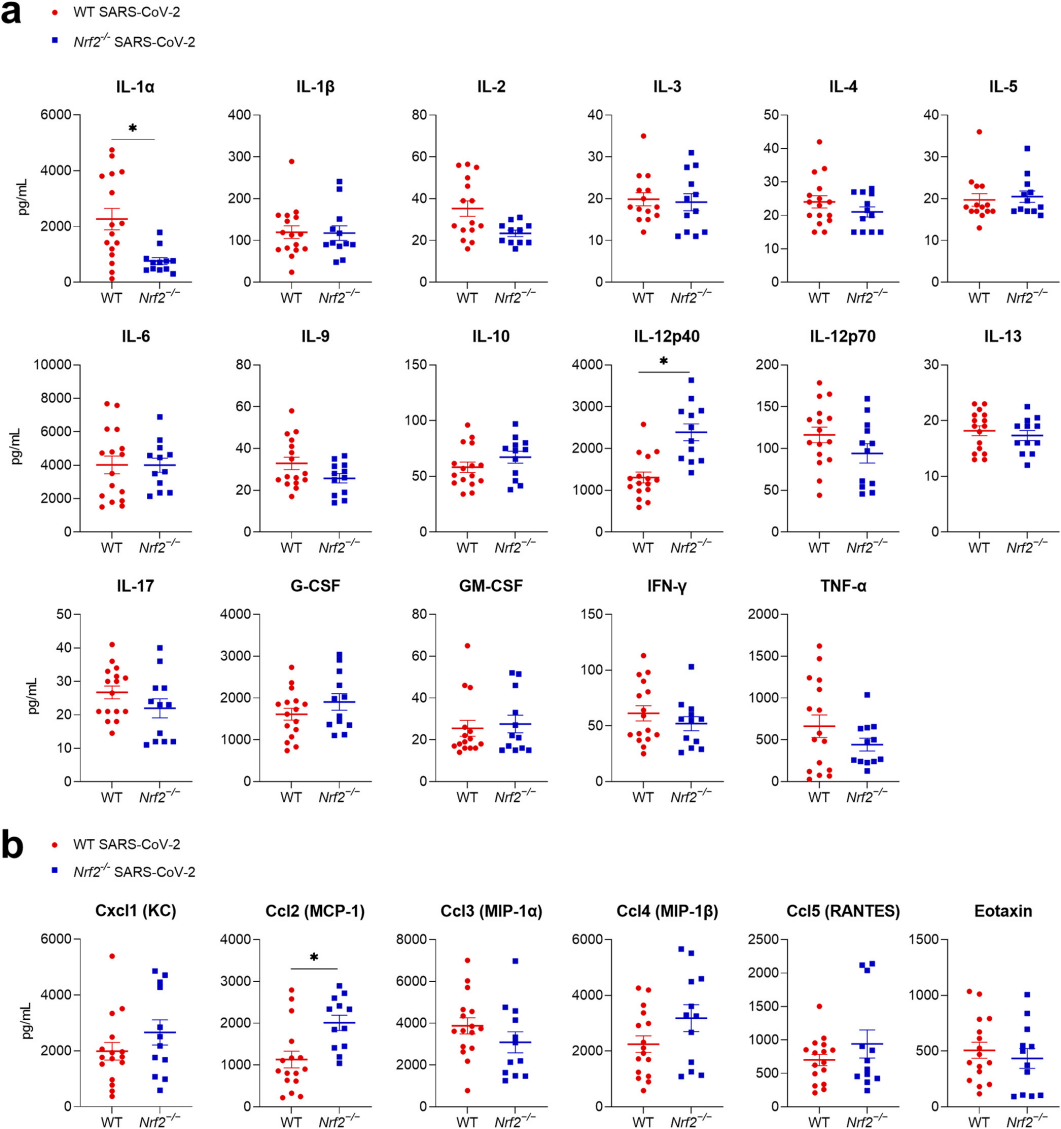
Cytokines and chemokines in response to SARS-CoV-2 infection in wild-type (WT) versus *Nrf2^−/−^* mice. Sixteen- to 20-week-old BALB/c WT and *Nrf2^−/−^* female mice were infected with 10^6^ TCID_50_s of mouse-adapted SARS-CoV-2 (CMA3p20) or mock inoculated. Bronchoalveolar lavage fluid (BALF) was collected at 2 dpi, and (a) cytokine and (b) chemokine levels were determined by using a Bio-Plex (23-plex assay). Data are expressed as mean ± SEM (mock, *n *= 3 mice/group [not shown but included in the statistical analysis]; SARS-CoV-2, *n *= 12 to 16 mice/group; *, *P* < 0.05 for WT SARS-CoV-2 versus *Nrf2^−/−^* SARS-CoV-2 by two-way ANOVA followed by Tukey’s test).

Cytokines and chemokines play an important immunoregulatory role and contribute to disease severity in SARS-CoV-2 infections (33). Therefore, to further understand the role of NRF2 in the regulation of SARS-CoV-2-induced inflammatory response and severity of disease, we measured levels of cytokines and chemokines in the bronchoalveolar lavage fluid (BALF) samples from *Nrf2^−/−^* and WT mice collected at 2 dpi (Fig. 6a and b). The *Nrf2^−/−^* mice exhibited significantly increased secretion of interleukin-12p40 (IL-12p40) and chemokine (C-C motif) ligand 2 (Ccl2)/monocyte chemoattractant protein-1 (MCP-1), while displaying decreased secretion of IL-1α, compared to infected WT mice. There were no significant changes between those groups in the other 20 cytokines and chemokines analyzed.

The virus-induced oxidative stress also contributes to disease severity (34). To assess whether increased oxidative stress could be related to the observed exacerbated disease in the *Nrf2^−/−^* mice, lung oxidative stress was estimated in BALF of the mice by measuring a marker of protein oxidation. As expected, after SARS-CoV-2 infection, there was a significant increase in the content of advanced oxidation protein products (AOPPs) in the BALF of mice, indicating increased oxidative stress in the lungs after infection (Fig. S2). However, the lack of *Nrf2* did not result in worsening oxidative stress at 2 dpi, suggesting that the increased inflammatory response is more likely related to the increased disease severity observed in the *Nrf2*-deficient mice.

## DISCUSSION

In this study, we showed that SARS-CoV-2—both the early pandemic viral strain and the more recent Omicron variant—strongly reduced the expression levels of NRF2 protein in multiple cell lines, including lung-derived epithelial cells. This effect was previously shown in Vero cells by Zhang et al. (35), and our data extended the observation to one of the Omicron strains, indicating that new variants also have the potential to interfere with important innate pathways that control the antioxidant and cytoprotective machinery of the infected host cell. Of clinical relevance, our study demonstrated for the first time that SARS-CoV-2 inhibits NRF2 expression in human lung epithelial cells, which are more relevant cell models for SARS-CoV-2 infection.

One question that was addressed in this study is the possible mechanism(s) responsible for the observed decrease in NRF2 cellular levels in the infected cells, which is currently unknown. NRF2 is normally bound in the cytosol to an inhibitor known as KEAP1. This association renders NRF2 inactive by shuttling it toward degradation through the ubiquitin-proteasome pathway (16). In previous studies by our group, we have shown that infection with another human viral pathogen, RSV, leads to increased NRF2 ubiquitination and degradation through the proteasomal pathway (30). However, in this study, our results using a proteasome inhibitor excluded the involvement of this pathway in SARS-CoV-2-infected cells. In addition to KEAP1, other cellular pathways have been identified in controlling NRF2 degradation. Among them, it has been shown that NRF2 may undergo SUMOylation in the nucleus and become a substrate of the RNF4, a small ubiquitin-like modifier (SUMO)-specific E3 ubiquitin ligase targeting NRF2 for degradation (36). PML protein is a member of the TRIM family and a major component of PML NBs. PML and NBs are involved in a wide variety of cellular processes, through facilitation of posttranslational modifications of partner proteins (notably SUMOylation), resulting in partner sequestration, activation, or degradation (37). PML is primarily upregulated in infection by type I and type II IFNs (38, 39). In previous studies, we found that this pathway is involved in the RSV-induced NRF2 degradation, since RSV infection induces the expression of PML protein and PML NB formation in an IFN-dependent manner and also induces NRF2-PML NB association (31). The results of this study seem to exclude an involvement of this pathway, primarily because SARS-CoV-2 infection, in stark contrast with RSV, did not upregulate PML cellular levels. As mentioned, PML protein is IFN induced and SARS-CoV-2 appears to be a poor inducer of type I and type III IFN responses (40), including in studies where it has been tested side by side with RSV (41). Moreover, another indication that the mechanism(s) involved in the NRF2 downregulation by SARS-CoV-2 is IFN/PML independent is the observation that it also occurs in Vero cells, which are known to have lost the ability to produce IFN (42). Overall, based on our studies both proteasomal degradation and the IFN/PML pathway appear to be dispensable for SARS-CoV-2-mediated downregulation of cellular NRF2. Additional investigations will be necessary to identify the mechanism(s) of NRF2 degradation.

Our study demonstrates not only that SARS-CoV-2 reduces the expression of NRF2 in cells, but it also affects its biological transcriptional activity as the infection resulted in a significant decrease in the expression of a cluster of NRF2-dependent genes—*SOD1*, *CAT*, *GPX1*, *GCLC*, *NQO1*, and *HMOX1*—in human lung-derived epithelial cells. These gene products play an important role in protecting against oxidative stress and lung tissue damage, thus suggesting that this global inhibitory process of AOE may play an important pathogenetic role in SARS-CoV-2 infections. In this context, Olagnier et al. (25) reported that the expression of NRF2-inducible proteins HMOX1 and NQO1 was repressed in SARS-CoV-2-infected Vero-TMPRSS2 cells. Zhang et al. (35) also showed that HMOX1 protein expression was suppressed by SARS-CoV-2 in Vero E6 cells and *HMOX1* and *NQO1* gene expression was reduced in human liver-derived Huh7 cells after SARS-CoV-2 infection. In addition, Bartolini et al. (43) showed that SARS-CoV-2 infection impaired the metabolism of cellular glutathione, by lowering the reduced form of glutathione (GSH) and increasing the levels of oxidized glutathione (GSSG) in Vero E6 cells. They showed that GCLC protein levels were initially increased at 6 hpi but decreased at 24 hpi. In contrast, the expression of the proteins NQO1 and GSTP was found to be upregulated at 24 hpi (43).

The importance of our findings in cell models was corroborated by the studies that we performed in mouse models of SARS-CoV-2 infection. For those, we used a mouse-adapted strain of SARS-CoV-2 (CMA3p20) which has been generated by a reverse-genetics system (32). By incorporating key mutations found in SARS-CoV-2 variants, the CMA3p20 virus recapitulates critical elements of human infection, including viral replication in the lung and disease. Importantly, mouse adaptation of SARS-CoV-2 does not impair replication in human airway cells and maintains antigenicity similar to human SARS-CoV-2 strains (32). Overall, CMA3p20 infection of BALB/c mice caused a significant decrease in the expression of the AOE genes in the lung tissue, specifically *Cat*, *Gstm1*, and *Prdx6*, while it did not affect *Sod1* and *Prdx1*. Importantly, our findings in mouse lungs parallel published data from a study of human lung biopsy specimens in which expression of NRF2-driven genes was found to be suppressed in COVID-19 patients compared to control biopsy specimens (25). Different from what we observed in infected human epithelial cells, we found that the *Hmox1* gene was upregulated by SARS-CoV-2 infection in mouse lung at 2 dpi. In addition to NRF2, other signaling molecules are known to regulate *HMOX1* gene transcription, including NF-κB, the important mediator of the immune and inflammatory responses (44, 45). It also possible that the *HMOX1* gene could be downregulated in airway epithelial cells but upregulated in other types of cells, such as the ones involved in the inflammatory response in the lung, which are present in the infected area from which RNA is extracted for RT-qPCR analysis.

To further assess the role of NRF2 in SARS-CoV-2 infection, we performed studies in mice genetically deficient in *Nrf2* (*Nrf2^−/−^*). These are the first studies to the best of our knowledge that have been performed with SARS-CoV-2 in this mouse model. Our results showed that the *Nrf2* deficiency in mice was associated with exacerbated clinical disease, as manifested by greater and prolonged body weight loss compared to control WT mice. In addition, the *Nrf2^−/−^* mice showed an increase in the lung inflammatory response following SARS-CoV-2 infection compared to infected WT mice. They had significantly higher secretion of IL-12p40 and MCP-1 in the BALF at 2 dpi, as well as increased peribronchial inflammation at 7 dpi, which suggests that NRF2 contributes to the “protection” of the airways during SARS-CoV-2 infection. Interestingly, the infected *Nrf2^−/−^* mice showed lower secretion of IL-1α in the BALF at 2 dpi than the infected WT mice. The IL-1α gene expression was reported to be upregulated in the lungs of *Nrf2^−/−^* mice 30 min after lipopolysaccharide (LPS) challenge (46), and there was no change in a model of pneumovirus infection (47). A more inclusive kinetics, with earlier time points of infection, might clarify this discrepancy.

No significant differences in collagen deposition in the lung were observed at 7 dpi between SARS-CoV-2-infected *Nrf2^−/−^* and WT mice, although we observed a trend toward higher percentage of collagen in the *Nrf2^−/−^* mice. We need to point out that 7 dpi is likely to be quite early to detect signs of airway fibrosis following infection. By using a different mouse-adapted strain of SARS-CoV-2 (MA10), Dinnon et al. (48) found higher profibrotic disease/collagen deposition starting at 15 to 120 dpi in aged BALB/c mice. Other respiratory viruses, including RSV and hMPV, have been tested in mice deficient for *Nrf2*, and those studies in general have shown that lack of *Nrf2* is associated with enhanced disease, lung pathology, and cytokine-driven inflammation, alteration of parameters of airway function, and skewed T cell immunity (47, 49). One study has also reported increased collagen deposition in *Nrf2^−/−^* neonatal mice exposed to a combination of hyperoxia and RSV infection (50).

Although it did not reach statistical significance, we found a trend for higher peak SARS-CoV-2 titers in mouse lung of *Nrf2^−/−^* mice compared to infected WT mice. This finding is similar to what has been reported by us and others in other models of viral respiratory pathogens in *Nrf2^−/−^* mice (47, 49). The mechanisms underlying the increased viral replication in the lung of *Nrf2^−/−^* mice are still unclear. The relative defect in the antioxidant defense system in the absence of NRF2 could in part explain this finding. In previous work, treatment of airway epithelial cells with either of the salen-manganese complexes EUK-8 and EUK-189, which possess SOD, CAT, and GPX activity, strongly reduced RSV-induced ROS formation by increasing cellular AOE enzymatic activity and reduced viral replication (51). In experimental infections with influenza virus or RSV, exogenous treatment with the AOE CAT significantly reduced viral titers in the lung of mice (52, 53). In addition, NRF2 controls the expression of cystathionine-γ-lyase (CSE) in airway epithelial cells (54), which is a critical enzyme for the biosynthesis of hydrogen sulfide, which has a broad antiviral activity (55–57) and whose expression was decreased in RSV-infected *Nrf2^−/−^* mice compared to WT mice (47). NRF2 has also been shown to affect innate immunity, including macrophage and dendritic cell antigen-presenting functions, and T helper cell balance (i.e., favoring Th1 responses), while oxidative stress might be involved in the loss of naive T cells and decrease in Th1 immunity (46, 58). More studies will be necessary to understand if the antiviral response and the mechanisms that controls viral replication in the lung are affected by the NRF2-mediated gene network.

In conclusion, by using relevant human airway epithelial cells and mouse models our study provides mechanistic evidence supporting current clinical evidence of a role for oxidative stress in the pathogenesis of COVID-19. In this context, we show that NRF2 plays an important function as a key regulator of the antioxidant pathway in SARS-CoV-2 infection, particularly in the airway epithelium and in the lung. Future studies with SARS-CoV-2 infection will address virus-specific mechanisms that control NRF2 downregulation in cells and will expand the breath of investigations addressing the role of the NRF2-dependent gene network in antiviral responses, innate and adaptive immunity, and chronic airway remodeling and the effect of senescence on such critical antiviral pathway.

## MATERIALS AND METHODS

### Biosafety.

All experiments involving infectious SARS-CoV-2 were conducted in biosafety level 3 (BSL3) laboratories at The University of Texas Medical Branch (UTMB) at Galveston, TX, USA.

### Ethics statement.

All procedures involving mice were performed in accordance with the recommendations in the *Guide for the Care and Use of Laboratory Animals* (59) of the National Institutes of Health. The studies were approved by the Institutional Animal Care and Use Committee (IACUC) of the UTMB at Galveston TX (protocols 2102014 and 9001002).

### Cells and reagents.

Vero E6 cells were grown in high-glucose Dulbecco’s modified Eagle’s medium (DMEM) supplemented with 10% fetal bovine serum (FBS), 100 mg/L sodium pyruvate, 100,000 U/L penicillin-streptomycin (P/S), and 25 mM HEPES. Vero cells stably expressing human transmembrane serine protease 2 (Vero-TMPRSS2) were grown in low-glucose DMEM supplemented with 10% FBS, 2% Geneticin selective antibiotic (G418 sulfate), 100,000 U/L P/S, and 25 mM HEPES. A549 cells that stably express human ACE2 receptor (A549-hACE2) were grown in high-glucose DMEM supplemented with 10% FBS, 1% P/S, 1% HEPES, and 10 μg/mL blasticidin S. Vero-TMPRSS2 and A549-hACE2 cells were kindly provided by Pei-Yong Shi. Calu-3 cells were grown in low-glucose DMEM supplemented with 10% FBS, 1× antibiotic/antimycotic, and 1 mg/mL sodium pyruvate. Calu-3 cells were differentiated at the ALI for several weeks before use. All cell lines were maintained at 37°C with 5% CO_2_. Cell culture media, supplements, and antibiotics were purchased from Thermo Fisher Scientific (Waltham, MA, USA). Lactacystin (catalog no. 426100) was purchased from Sigma-Aldrich (St. Louis, MO, USA), and human IFN-β (part no. SMP146) was purchased from PBL Assay Science (Piscataway, NJ, USA).

### Viruses and *in vitro* infection.

The SARS-CoV-2 USA-WA1/2020 strain was propagated in Vero E6 cells in high-glucose Dulbecco’s modified Eagle’s medium (DMEM) supplemented with 5% FBS, 100 mg/L sodium pyruvate, 100,000 U/L penicillin-streptomycin (P/S), and 25 mM HEPES. The SARS-CoV-2 Omicron variant (hCov/EHC_C19_2811C, lineage B.1.1.529) was propagated in Vero-TMPRSS2 cells in low-glucose DMEM supplemented with 5% FBS, 2% Geneticin selective antibiotic (G418 sulfate), 100,000 U/L P/S, and 25 mM HEPES. SARS-CoV-2 titers were determined by the 50% tissue culture infective dose (TCID_50_) assay and calculated according to the method of Reed and Muench (60) based on four replicates for dilution. The RSV Long strain was propagated in HEp-2 cells and purified by ultracentrifugation in a sucrose density gradient. The titer of RSV viral pools was determined by methylcellulose plaque assay and expressed as PFU per milliliter. The cells were infected with the SARS-CoV-2 variants at a multiplicity of infection (MOI) of 1.5 or 3 and with RSV at an MOI of 5 in medium without FBS for 1 to 1.5 h and then cultured with fresh medium supplemented with 5% FBS throughout the length of the experiments.

### Western blotting.

Cells were lysed directly in the cell culture plates with 3× SDS loading buffer (B7703S; New England BioLabs, Ipswich, MA, USA). After boiling at 95 to 100°C for 10 min, the samples were removed from the BSL3 facility for further processing. Cell lysates were briefly sonicated (10 to 12 s) to reduce sample viscosity. After centrifugation, supernatants were collected and boiled at 95 to 100°C for 5 min. An equal volume of samples (25 to 30 μL) was separated by SDS-PAGE and transferred onto a polyvinylidene difluoride (PVDF) membrane. Nonspecific binding was blocked by immersing the membrane in Tris-buffered saline-Tween (TBST) blocking solution containing 5% skim milk powder. After blocking, the membranes were incubated with the primary antibody overnight at 4°C followed by the appropriate secondary antibody for 1 h at room temperature. Proteins were detected using enhanced chemiluminescence (ECL). The primary antibodies used were anti-NRF2 (ab62352; Abcam, Cambridge, United Kingdom), anti-PML protein (ab179466; Abcam, Cambridge, United Kingdom), and anti-β-actin as the loading control (A1978 [Sigma-Aldrich, St. Louis, MO, USA] and sc-47778 HRP [Santa Cruz Biotechnology, Dallas, TX, USA]).

### RNA extraction and RT-qPCR.

In the *in vitro* experiments, total RNA was isolated from cells using TRIzol reagent (15596018; Thermo Fisher Scientific, Waltham, MA, USA). Synthesis of cDNA was performed with 1 μg of total RNA using iScript reverse transcription supermix (1708841; Bio-Rad Laboratories, Hercules, CA, USA). The cDNA was diluted five times, and quantitative PCR (qPCR) was done using 2 μL of cDNA, premixed forward and reverse primers and probe, and TaqMan Universal master mix (4440040; Applied Biosystems, Waltham, MA, USA). The qPCR assays were run in the Bio-Rad CFX Connect real-time system. The RNA from murine lungs was isolated using a combination of TRIzol-based method and the RNeasy minikit (74104; Qiagen, Hilden, Germany). Briefly, lung tissues were homogenized in TRIzol reagent (15596018; Thermo Fisher Scientific, Waltham, MA, USA) with stainless steel beads (7-mm diameter) using the TissueLyser LT (85600; Qiagen, Hilden, Germany). After 24 h in TRIzol, the samples were removed from the BSL3 facility for further processing. After the addition of chloroform, the clear aqueous phase was mixed with absolute ethanol and transferred to an RNeasy mini-spin column, followed by the washes recommended in the RNeasy minikit protocol. On-column DNase digestion was performed using the RNase-free DNase set (79254; Qiagen, Hilden, Germany). Synthesis of cDNA was performed with 1 μg of total RNA using TaqMan reverse transcription reagents (N8080234; Applied Biosystems, Waltham, MA, USA). The amplification was done with 1 μL of cDNA in a total reaction volume of 20 μL using the iTaq Universal SYBR green supermix (1725124; Bio-Rad Laboratories, Hercules, CA, USA). The qPCR assays were run in the ABI Prism 7500 sequence detection system. The 18S rRNA was used as a housekeeping gene for normalization. The threshold cycle (2^−ΔΔ^*^CT^*) method was used to calculate relative quantification. Human *SOD1*, *CAT*, *GPX1*, *GCLC*, *NQO1*, and *HMOX1* and mouse *Sod1*, *Cat*, *Gstm1*, *Prdx1*, *Prdx6*, and *Hmox1* primer sequences are available upon request.

### Mouse-adapted SARS-CoV-2 strain.

The mouse-adapted strain of SARS-CoV-2 CMA3p20 (32) was propagated in Vero E6 cells in DMEM supplemented with 5% FBS, 4.5 g/L d-glucose, 100 mg/L sodium pyruvate, 100,000 U/L P/S, and 25 mM HEPES. Viral titers, expressed as TCID_50_ per milliliter, were calculated according to Reed and Muench (60) based on four replicates for dilution.

### Mice and infection protocol.

Experiments for body weight loss and gene expression analyses were performed using 11- to 12-week-old BALB/c female mice purchased from The Jackson Laboratory (Bar Harbor, ME, USA). *Nrf2^−/−^* mice on a mixed C57BL/6 and AKR background were generated as previously described (61) and received as a generous gift from Jefferson Chan at the University of California San Francisco and Karen T. Liby at the Michigan State University. These mice were backcrossed onto a BALB/c background for eight generations and were found to be 99% congenic (analysis performed by The Jackson Laboratory). A pathogen-free breeding colony of *Nrf2^−/−^* mice was maintained at UTMB, Galveston, TX. Experiments were performed using 16- to 20-week-old *Nrf2^−/−^* and WT age-matched female mice. Prior to infection, mice were anesthetized with isoflurane and infected intranasally with 10^6^ TCID_50_ of the mouse-adapted strain of SARS-CoV-2 (CMA3p20) diluted in 50 μL of phosphate-buffered saline (PBS) or mock inoculated with the same volume of PBS. The mice were monitored daily for body weight loss, a well-established parameter of clinical disease in mice, and were euthanized at 2, 3, or 7 dpi.

### Bronchoalveolar lavage fluid.

Bronchoalveolar lavage fluid (BALF) was collected *in situ* by flushing the lungs via the trachea twice with 1 mL of ice-cold PBS.

### SARS-CoV-2 titration of lung tissue.

Lung viral titers were determined by TCID_50_ assay on 96-well plates (Vero E6 cells). After 3 days of incubation at 37°C, wells were scored for cytopathic effect (CPE). Viral titers, expressed as TCID_50_ per milliliter, were calculated according to the method of Reed and Muench (60) based on four replicates for dilution.

### Pulmonary histopathology.

The lungs of mice euthanized at 7 dpi were fixed with 10% neutral buffered formalin, embedded in paraffin, sectioned, and stained with H&E and Masson’s trichrome. The H&E-stained slides were analyzed under light microscopy by a board-certified pathologist with expertise in mouse lung (Y.L.J.-H.). Briefly, inflammatory infiltrates were scored by assessing the layers of inflammatory cells surrounding the vessels and bronchioles throughout the whole-lung section. Abnormality was judged as more than three layers of inflammatory cells surrounding 50% or more of the circumference of the vessel or bronchioles. A total of ~15 perivascular and peribronchial spaces were counted for each slide (62). The percentages of peribronchiolitis and perivasculitis were scored 0 to 3: 0 = normal, 1 = ~5 to 25%, 2 = 26 to 75%, and 3 = >75%. The percentages are approximate based on visual impression by the study pathologist. For determination of the percentage of collagen in lung tissue, slides were scanned at ×20 magnification using the Pannoramic Scan II by 3DHistec, and images were analyzed using the Visiopharm software (v.2022.01). A custom Visiopharm APP was developed to assess the percentage of blue staining in the lung slides (representing collagen). The collagen ratio was determined by dividing the area of collagen staining by the total tissue area evaluated (region of interest). This number was multiplied by 100 to determine the percentage of collagen per tissue (63, 64).

### Cytokine and chemokine analysis.

Levels of cytokines and chemokines were determined in BALF of mice using the Bio-Plex Pro mouse cytokine 23-plex assay (M60009RDPD; Bio-Rad Laboratories, Hercules, CA, USA) according to the manufacturer’s protocol.

### Advanced oxidation protein products.

Advanced oxidation protein products (AOPPs), a marker of oxidative stress, were measured in BALF of mice using the OxiSelect AOPP assay kit (STA-318; Cell Biolabs, San Diego, CA, USA) following the manufacturer’s instructions. The AOPP content was determined by comparison with a chloramine standard curve and expressed in micromolar concentration chloramine equivalent units.

### Statistical analysis.

The data were analyzed using GraphPad Prism 9. The specifics of statistical comparisons are detailed in the figure legends. Results are expressed as the mean ± standard error of the mean (SEM), and a *P* value of <0.05 was considered statistically significant.

## References

[1] Lee C. 2018. Therapeutic modulation of virus-induced oxidative stress via the Nrf2-dependent antioxidative pathway. Oxid Med Cell Longev 2018:6208067. doi:10.1155/2018/6208067. 30515256 PMC6234444

[2] Hosakote YM, Liu T, Castro SM, Garofalo RP, Casola A. 2009. Respiratory syncytial virus induces oxidative stress by modulating antioxidant enzymes. Am J Respir Cell Mol Biol 41:348–357. doi:10.1165/rcmb.2008-0330OC. 19151318 PMC2742754

[3] Liu M, Chen F, Liu T, Chen F, Liu S, Yang J. 2017. The role of oxidative stress in influenza virus infection. Microbes Infect 19:580–586. doi:10.1016/j.micinf.2017.08.008. 28918004

[4] Komaravelli N, Kelley JP, Garofalo MP, Wu H, Casola A, Kolli D. 2015. Role of dietary antioxidants in human metapneumovirus infection. Virus Res 200:19–23. doi:10.1016/j.virusres.2015.01.018. 25645280 PMC5022781

[5] Komaravelli N, Casola A. 2014. Respiratory viral infections and subversion of cellular antioxidant defenses. J Pharmacogenomics Pharmacoproteomics 5:1000141. doi:10.4172/2153-0645.1000141. 25584194 PMC4288774

[6] Pham-Huy LA, He H, Pham-Huy C. 2008. Free radicals, antioxidants in disease and health. Int J Biomed Sci 4:89–96. 23675073 PMC3614697

[7] Itoh K, Ishii T, Wakabayashi N, Yamamoto M. 1999. Regulatory mechanisms of cellular response to oxidative stress. Free Radic Res 31:319–324. doi:10.1080/10715769900300881. 10517536

[8] Kurutas EB. 2015. The importance of antioxidants which play the role in cellular response against oxidative/nitrosative stress: current state. Nutr J 15:71. doi:10.1186/s12937-016-0186-5. PMC4960740 27456681

[9] Erlich JR, To EE, Liong S, Brooks R, Vlahos R, O'Leary JJ, Brooks DA, Selemidis S. 2020. Targeting evolutionary conserved oxidative stress and immunometabolic pathways for the treatment of respiratory infectious diseases. Antioxid Redox Signal 32:993–1013. doi:10.1089/ars.2020.8028. 32008371 PMC7426980

[10] Tonelli C, Chio IIC, Tuveson DA. 2018. Transcriptional regulation by Nrf2. Antioxid Redox Signal 29:1727–1745. doi:10.1089/ars.2017.7342. 28899199 PMC6208165

[11] Herengt A, Thyrsted J, Holm CK. 2021. NRF2 in viral infection. Antioxidants (Basel) 10:1491. doi:10.3390/antiox10091491. 34573123 PMC8472116

[12] Ramezani A, Nahad MP, Faghihloo E. 2018. The role of Nrf2 transcription factor in viral infection. J Cell Biochem 119:6366–6382. doi:10.1002/jcb.26897. 29737559

[13] Dodson M, Castro-Portuguez R, Zhang DD. 2019. NRF2 plays a critical role in mitigating lipid peroxidation and ferroptosis. Redox Biol 23:101107. doi:10.1016/j.redox.2019.101107. 30692038 PMC6859567

[14] Jung KA, Kwak MK. 2010. The Nrf2 system as a potential target for the development of indirect antioxidants. Molecules 15:7266–7291. doi:10.3390/molecules15107266. 20966874 PMC6259123

[15] Vomund S, Schafer A, Parnham MJ, Brune B, von Knethen A. 2017. Nrf2, the master regulator of anti-oxidative responses. Int J Mol Sci 18:2772. doi:10.3390/ijms18122772. 29261130 PMC5751370

[16] Kaspar JW, Niture SK, Jaiswal AK. 2009. Nrf2:INrf2 (Keap1) signaling in oxidative stress. Free Radic Biol Med 47:1304–1309. doi:10.1016/j.freeradbiomed.2009.07.035. 19666107 PMC2763938

[17] Cuadrado A, Pajares M, Benito C, Jiménez-Villegas J, Escoll M, Fernández-Ginés R, Garcia Yagüe AJ, Lastra D, Manda G, Rojo AI, Dinkova-Kostova AT. 2020. Can activation of NRF2 be a strategy against COVID-19? Trends Pharmacol Sci 41:598–610. doi:10.1016/j.tips.2020.07.003. 32711925 PMC7359808

[18] Khomich OA, Kochetkov SN, Bartosch B, Ivanov AV. 2018. Redox biology of respiratory viral infections. Viruses 10:392. doi:10.3390/v10080392. 30049972 PMC6115776

[19] Checconi P, De Angelis M, Marcocci ME, Fraternale A, Magnani M, Palamara AT, Nencioni L. 2020. Redox-modulating agents in the treatment of viral infections. Int J Mol Sci 21:4084. doi:10.3390/ijms21114084. 32521619 PMC7312898

[20] Delgado-Roche L, Mesta F. 2020. Oxidative stress as key player in severe acute respiratory syndrome coronavirus (SARS-CoV) infection. Arch Med Res 51:384–387. doi:10.1016/j.arcmed.2020.04.019. 32402576 PMC7190501

[21] Cecchini R, Cecchini AL. 2020. SARS-CoV-2 infection pathogenesis is related to oxidative stress as a response to aggression. Med Hypotheses 143:110102. doi:10.1016/j.mehy.2020.110102. 32721799 PMC7357498

[22] Suhail S, Zajac J, Fossum C, Lowater H, McCracken C, Severson N, Laatsch B, Narkiewicz-Jodko A, Johnson B, Liebau J, Bhattacharyya S, Hati S. 2020. Role of oxidative stress on SARS-CoV (SARS) and SARS-CoV-2 (COVID-19) infection: a review. Protein J 39:644–656. doi:10.1007/s10930-020-09935-8. 33106987 PMC7587547

[23] Yildiz H, Alp HH, Ekin S, Arisoy A, Gunbatar H, Asker S, Cilingir BM, Sunnetcioglu A, Celikel M, Esen N, Bedirhanoglu S, Baykal ND, Haylu M. 2021. Analysis of endogenous oxidative damage markers and association with pulmonary involvement severity in patients with SARS-CoV-2 pneumonia. Infect Dis Now 51:429–434. doi:10.1016/j.idnow.2021.06.302. 34146758 PMC8236077

[24] Saheb Sharif-Askari N, Saheb Sharif-Askari F, Mdkhana B, Hussain Alsayed HA, Alsafar H, Alrais ZF, Hamid Q, Halwani R. 2021. Upregulation of oxidative stress gene markers during SARS-COV-2 viral infection. Free Radic Biol Med 172:688–698. doi:10.1016/j.freeradbiomed.2021.06.018. 34186206 PMC8233550

[25] Olagnier D, Farahani E, Thyrsted J, Blay-Cadanet J, Herengt A, Idorn M, Hait A, Hernaez B, Knudsen A, Iversen MB, Schilling M, Jørgensen SE, Thomsen M, Reinert LS, Lappe M, Hoang HD, Gilchrist VH, Hansen AL, Ottosen R, Nielsen CG, Møller C, van der Horst D, Peri S, Balachandran S, Huang J, Jakobsen M, Svenningsen EB, Poulsen TB, Bartsch L, Thielke AL, Luo Y, Alain T, Rehwinkel J, Alcamí A, Hiscott J, Mogensen TH, Paludan SR, Holm CK. 2020. SARS-CoV2-mediated suppression of NRF2-signaling reveals potent antiviral and anti-inflammatory activity of 4-octyl-itaconate and dimethyl fumarate. Nat Commun 11:4938. doi:10.1038/s41467-020-18764-3. 33009401 PMC7532469

[26] Leist SR, Schäfer A, Martinez DR. 2020. Cell and animal models of SARS-CoV-2 pathogenesis and immunity. Dis Model Mech 13:dmm046581. doi:10.1242/dmm.046581. 32887790 PMC7490513

[27] Johnson BA, Zhou Y, Lokugamage KG, Vu MN, Bopp N, Crocquet-Valdes PA, Kalveram B, Schindewolf C, Liu Y, Scharton D, Plante JA, Xie X, Aguilar P, Weaver SC, Shi PY, Walker DH, Routh AL, Plante KS, Menachery VD. 2022. Nucleocapsid mutations in SARS-CoV-2 augment replication and pathogenesis. PLoS Pathog 18:e1010627. doi:10.1371/journal.ppat.1010627. 35728038 PMC9275689

[28] Mossel EC, Huang C, Narayanan K, Makino S, Tesh RB, Peters CJ. 2005. Exogenous ACE2 expression allows refractory cell lines to support severe acute respiratory syndrome coronavirus replication. J Virol 79:3846–3850. doi:10.1128/JVI.79.6.3846-3850.2005. 15731278 PMC1075706

[29] Lokugamage KG, Hage A, de Vries M, Valero-Jimenez AM, Schindewolf C, Dittmann M, Rajsbaum R, Menachery VD. 2020. Type I interferon susceptibility distinguishes SARS-CoV-2 from SARS-CoV. J Virol 94:e01410-20. doi:10.1128/JVI.01410-20. PMC7654262 32938761

[30] Komaravelli N, Tian B, Ivanciuc T, Mautemps N, Brasier AR, Garofalo RP, Casola A. 2015. Respiratory syncytial virus infection down-regulates antioxidant enzyme expression by triggering deacetylation-proteasomal degradation of Nrf2. Free Radic Biol Med 88:391–403. doi:10.1016/j.freeradbiomed.2015.05.043. 26073125 PMC4628892

[31] Komaravelli N, Ansar M, Garofalo RP, Casola A. 2017. Respiratory syncytial virus induces NRF2 degradation through a promyelocytic leukemia protein-ring finger protein 4 dependent pathway. Free Radic Biol Med 113:494–504. doi:10.1016/j.freeradbiomed.2017.10.380. 29107745 PMC5699968

[32] Muruato A, Vu MN, Johnson BA, Davis-Gardner ME, Vanderheiden A, Lokugamage K, Schindewolf C, Crocquet-Valdes PA, Langsjoen RM, Plante JA, Plante KS, Weaver SC, Debbink K, Routh AL, Walker D, Suthar MS, Shi PY, Xie X, Menachery VD. 2021. Mouse-adapted SARS-CoV-2 protects animals from lethal SARS-CoV challenge. PLoS Biol 19:e3001284. doi:10.1371/journal.pbio.3001284. 34735434 PMC8594810

[33] Hsu RJ, Yu WC, Peng GR, Ye CH, Hu S, Chong PCT, Yap KY, Lee JYC, Lin WC, Yu SH. 2022. The role of cytokines and chemokines in severe acute respiratory syndrome coronavirus 2 infections. Front Immunol 13:832394. doi:10.3389/fimmu.2022.832394. 35464491 PMC9021400

[34] Balakrishna Pillai A, JeanPierre AR, Mariappan V, Ranganadin P, Rao SR. 2022. Neutralizing the free radicals could alleviate the disease severity following an infection by positive strand RNA viruses. Cell Stress Chaperones 27:189–195. doi:10.1007/s12192-022-01269-x. 35366756 PMC8976658

[35] Zhang S, Wang J, Wang L, Aliyari S, Cheng G. 2022. SARS-CoV-2 virus NSP14 impairs NRF2/HMOX1 activation by targeting Sirtuin 1. Cell Mol Immunol 19:872–882. doi:10.1038/s41423-022-00887-w. 35732914 PMC9217730

[36] Malloy MT, McIntosh DJ, Walters TS, Flores A, Goodwin JS, Arinze IJ. 2013. Trafficking of the transcription factor Nrf2 to promyelocytic leukemia-nuclear bodies: implications for degradation of NRF2 in the nucleus. J Biol Chem 288:14569–14583. doi:10.1074/jbc.M112.437392. 23543742 PMC3656310

[37] Lallemand-Breitenbach V, de Thé H. 2010. PML nuclear bodies. Cold Spring Harb Perspect Biol 2:a000661. doi:10.1101/cshperspect.a000661. 20452955 PMC2857171

[38] Stadler M, Chelbi-Alix MK, Koken MH, Venturini L, Lee C, Saïb A, Quignon F, Pelicano L, Guillemin MC, Schindler C. 1995. Transcriptional induction of the PML growth suppressor gene by interferons is mediated through an ISRE and a GAS element. Oncogene 11:2565–2573. 8545113

[39] Heuser M, van der Kuip H, Falini B, Peschel C, Huber C, Fischer T. 1998. Induction of the pro-myelocytic leukaemia gene by type I and type II interferons. Mediators Inflamm 7:319–325. doi:10.1080/09629359890839. 9883966 PMC1781861

[40] Blanco-Melo D, Nilsson-Payant BE, Liu WC, Uhl S, Hoagland D, Møller R, Jordan TX, Oishi K, Panis M, Sachs D, Wang TT, Schwartz RE, Lim JK, Albrecht RA, tenOever BR. 2020. Imbalanced host response to SARS-CoV-2 drives development of COVID-19. Cell 181:1036–1045.e9. doi:10.1016/j.cell.2020.04.026. 32416070 PMC7227586

[41] Rajan A, Weaver AM, Aloisio GM, Jelinski J, Johnson HL, Venable SF, McBride T, Aideyan L, Piedra FA, Ye X, Melicoff-Portillo E, Yerramilli MRK, Zeng XL, Mancini MA, Stossi F, Maresso AW, Kotkar SA, Estes MK, Blutt S, Avadhanula V, Piedra PA. 2021. The human nose organoid respiratory virus model: an ex vivo human challenge model to study respiratory syncytial virus (RSV) and severe acute respiratory syndrome coronavirus 2 (SARS-CoV-2) pathogenesis and evaluate therapeutics. mBio 13:e03511-21. doi:10.1128/mbio.03511-21. 35164569 PMC8844923

[42] Emeny JM, Morgan MJ. 1979. Regulation of the interferon system: evidence that Vero cells have a genetic defect in interferon production. J Gen Virol 43:247–252. doi:10.1099/0022-1317-43-1-247. 113494

[43] Bartolini D, Stabile AM, Bastianelli S, Giustarini D, Pierucci S, Busti C, Vacca C, Gidari A, Francisci D, Castronari R, Mencacci A, Di Cristina M, Focaia R, Sabbatini S, Rende M, Gioiello A, Cruciani G, Rossi R, Galli F. 2021. SARS-CoV2 infection impairs the metabolism and redox function of cellular glutathione. Redox Biol 45:102041. doi:10.1016/j.redox.2021.102041. 34146958 PMC8190457

[44] Medina MV, Sapochnik D, Garcia Solá M, Coso O. 2020. Regulation of the expression of heme oxygenase-1: signal transduction, gene promoter activation, and beyond. Antioxid Redox Signal 32:1033–1044. doi:10.1089/ars.2019.7991. 31861960 PMC7153632

[45] Alam J, Cook JL. 2007. How many transcription factors does it take to turn on the heme oxygenase-1 gene? Am J Respir Cell Mol Biol 36:166–174. doi:10.1165/rcmb.2006-0340TR. 16990612

[46] Thimmulappa RK, Lee H, Rangasamy T, Reddy SP, Yamamoto M, Kensler TW, Biswal S. 2006. Nrf2 is a critical regulator of the innate immune response and survival during experimental sepsis. J Clin Invest 116:984–995. doi:10.1172/JCI25790. 16585964 PMC1421348

[47] Ivanciuc T, Sbrana E, Casola A, Garofalo RP. 2018. Protective role of nuclear factor erythroid 2-related factor 2 against respiratory syncytial virus and human metapneumovirus infections. Front Immunol 9:854. doi:10.3389/fimmu.2018.00854. 29740449 PMC5925606

[48] Dinnon KH, Leist SR, Okuda K, Dang H, Fritch EJ, Gully KL, De la Cruz G, Evangelista MD, Asakura T, Gilmore RC, Hawkins P, Nakano S, West A, Schäfer A, Gralinski LE, Everman JL, Sajuthi SP, Zweigart MR, Dong S, McBride J, Cooley MR, Hines JB, Love MK, Groshong SD, VanSchoiack A, Phelan SJ, Liang Y, Hether T, Leon M, Zumwalt RE, Barton LM, Duval EJ, Mukhopadhyay S, Stroberg E, Borczuk A, Thorne LB, Sakthivel MK, Lee YZ, Hagood JS, Mock JR, Seibold MA, O'Neal WK, Montgomery SA, Boucher RC, Baric RS. 2022. SARS-CoV-2 infection produces chronic pulmonary epithelial and immune cell dysfunction with fibrosis in mice. Sci Transl Med 14:eabo5070. doi:10.1126/scitranslmed.abo5070. 35857635 PMC9273046

[49] Cho H-Y, Imani F, Miller-DeGraff L, Walters D, Melendi GA, Yamamoto M, Polack FP, Kleeberger SR. 2009. Antiviral activity of Nrf2 in a murine model of respiratory syncytial virus (RSV) disease. Am J Respir Crit Care Med 179:138–150. doi:10.1164/rccm.200804-535OC. 18931336 PMC2633060

[50] Cho HY, Miller-DeGraff L, Perrow LA, Gladwell W, Panduri V, Lih FB, Kleeberger SR. 2021. Murine neonatal oxidant lung injury: NRF2-dependent predisposition to adulthood respiratory viral infection and protection by maternal antioxidant. Antioxidants (Basel) 10:1874. doi:10.3390/antiox10121874. 34942977 PMC8698620

[51] Hosakote YM, Komaravelli N, Mautemps N, Liu T, Garofalo RP, Casola A. 2012. Antioxidant mimetics modulate oxidative stress and cellular signaling in airway epithelial cells infected with respiratory syncytial virus. Am J Physiol Lung Cell Mol Physiol 303:L991–L1000. doi:10.1152/ajplung.00192.2012. 23023968 PMC3532525

[52] Shi X, Shi Z, Huang H, Zhu H, Zhu H, Ju D, Zhou P. 2013. PEGylated human catalase elicits potent therapeutic effects on H1N1 influenza-induced pneumonia in mice. Appl Microbiol Biotechnol 97:10025–10033. doi:10.1007/s00253-013-4775-3. 23525936 PMC7079947

[53] Ansar M, Ivanciuc T, Garofalo RP, Casola A. 2020. Increased lung catalase activity confers protection against experimental RSV infection. Sci Rep 10:3653. doi:10.1038/s41598-020-60443-2. 32107411 PMC7046725

[54] Jamaluddin M, Haas de Mello A, Tapryal N, Hazra TK, Garofalo RP, Casola A. 2022. NRF2 regulates cystathionine gamma-lyase expression and activity in primary airway epithelial cells infected with respiratory syncytial virus. Antioxidants (Basel) 11:1582. doi:10.3390/antiox11081582. 36009301 PMC9405023

[55] Ivanciuc T, Sbrana E, Ansar M, Bazhanov N, Szabo C, Casola A, Garofalo RP. 2016. Hydrogen sulfide is an antiviral and antiinflammatory endogenous gasotransmitter in the airways. Role in respiratory syncytial virus infection. Am J Respir Cell Mol Biol 55:684–696. doi:10.1165/rcmb.2015-0385OC. 27314446 PMC5105180

[56] Li H, Ma Y, Escaffre O, Ivanciuc T, Komaravelli N, Kelley JP, Coletta C, Szabo C, Rockx B, Garofalo RP, Casola A. 2015. Role of hydrogen sulfide in paramyxovirus infections. J Virol 89:5557–5568. doi:10.1128/JVI.00264-15. 25740991 PMC4442521

[57] Bazhanov N, Escaffre O, Freiberg AN, Garofalo RP, Casola A. 2017. Broad-range antiviral activity of hydrogen sulfide against highly pathogenic RNA viruses. Sci Rep 7:41029. doi:10.1038/srep41029. 28106111 PMC5247713

[58] Kim HJ, Barajas B, Wang M, Nel AE. 2008. Nrf2 activation by sulforaphane restores the age-related decrease of T(H)1 immunity: role of dendritic cells. J Allergy Clin Immunol 121:1255–1261.e7. doi:10.1016/j.jaci.2008.01.016. 18325578 PMC3897785

[59] National Research Council. 2011. Guide for the care and use of laboratory animals, 8th ed. National Academies Press, Washington, DC.

[60] Reed LJ, Muench H. 1938. A simple method of estimating fifty per cent endpoints. Am J Epidemiol 27:493–497. doi:10.1093/oxfordjournals.aje.a118408.

[61] Chan K, Lu R, Chang JC, Kan YW. 1996. NRF2, a member of the NFE2 family of transcription factors, is not essential for murine erythropoiesis, growth, and development. Proc Natl Acad Sci USA 93:13943–13948. doi:10.1073/pnas.93.24.13943. 8943040 PMC19474

[62] Haeberle HA, Casola A, Gatalica Z, Petronella S, Dieterich HJ, Ernst PB, Brasier AR, Garofalo RP. 2004. IkappaB kinase is a critical regulator of chemokine expression and lung inflammation in respiratory syncytial virus infection. J Virol 78:2232–2241. doi:10.1128/jvi.78.5.2232-2241.2004. 14963119 PMC369265

[63] Riber-Hansen R, Vainer B, Steiniche T. 2012. Digital image analysis: a review of reproducibility, stability and basic requirements for optimal results. APMIS 120:276–289. doi:10.1111/j.1600-0463.2011.02854.x. 22429210

[64] Bertram CA, Klopfleisch R. 2017. The pathologist 2.0: an update on digital pathology in veterinary medicine. Vet Pathol 54:756–766. doi:10.1177/0300985817709888. 28578626

