# Supplementary material for: SARS-CoV-2 Inhibits NRF2-Mediated Antioxidant Responses in Airway Epithelial Cells and in the Lung of a Murine Model of Infection
Source: Microbiol Spectr. 2023 Apr 6;11(3):e00378-23. doi: 10.1128/spectrum.00378-23 (PMC10269779; doi:10.1128/spectrum.00378-23)
Supplement: Supplemental file 1 — Fig. S1 and S2. Download spectrum.00378-23-s0001.pdf, PDF file, 0.2 MB [file spectrum.00378-23-s0001.pdf]

Supplemental Material

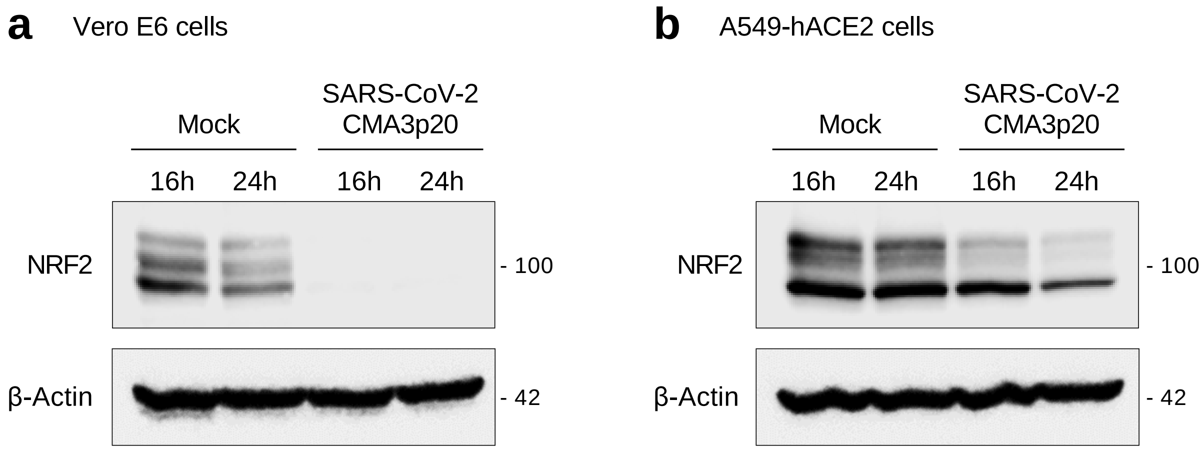

FIG S1 NRF2 protein levels in epithelial cell lines after infection with the mouse-adapted SARS-CoV-2 (CMA3p20). Vero E6 (a) and A549-hACE2 (b) cells were mock-infected or infected with SARS-CoV-2 CMA3p20 for 16 and 24 h. Whole cell lysates were analyzed by western blot with anti-NRF2 antibody. The membranes were reprobed for anti-β-actin antibody for loading control. Data from one experiment performed in duplicate.

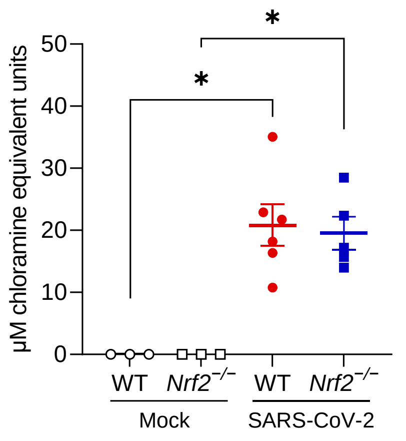

FIG S2 Marker of oxidative stress in the bronchoalveolar lavage fluid (BALF) in response to SARS-CoV-2 infection in wild type (WT) vs. *Nrf2*$^{-/-}$ mice. Sixteen- to twenty-week-old BALB/c WT and *Nrf2*$^{-/-}$ female mice were infected with 10^6 $TCID_{50}$ of mouse-adapted SARS-CoV-2 (CMA3p20) or mock-inoculated. BALF was collected 2 days postinfection and advanced oxidation protein products (AOPP) content was measured. Data are expressed as mean ± SEM (*n* = 3-6 mice/group, two-way ANOVA followed by Tukey's test, * $P < 0.05$).
